# Novel Iodine-induced Cleavage Real-time PCR Assay for Accurate Quantification of Phosphorothioate Modified Sites in Bacterial DNA

**DOI:** 10.1038/s41598-019-44011-x

**Published:** 2019-05-16

**Authors:** Yi Chen, Tao Zheng, Jinli Li, Jinjie Cui, Zixin Deng, Delin You, Litao Yang

**Affiliations:** 1 State Key Laboratory of Microbial Metabolism, Joint International Research Laboratory of Metabolic and Developmental Sciences, and School of Life Sciences & Biotechnology, Shanghai Jiao Tong University, Shanghai, 200030 China; 2 Institute of Cotton Research, Chinese Academy of Agricultural Sciences/State Key Laboratory of Cotton Biology, Anyang, Henan 455000 China

**Keywords:** Bacterial techniques and applications, Epigenomics

## Abstract

DNA Phosphorothioate (PT), replacing a non-bridging phosphate oxygen atom with a sulfur atom, is one kind of common DNA modification in bacteria. Whole genome scale description of the location and frequency of PT modification is the key to understand its biological function. Herein we developed a novel method, named with iodine-induced cleavage quantitative real-time PCR (IC-qPCR), to evaluate the frequency of PT modification at a given site in bacterial DNA. The efficiency, dynamic range, sensitivity, reproducibility and accuracy of IC-qPCR were well tested and verified employing an *E*. *coli B7A* strain as example. The amplification efficiency of IC-qPCR assay ranged from 91% to 99% with a high correlation coefficient ≥0.99. The limit of quantification was determined as low as 10 copies per reaction for the 607710 and 1818096 sites, and 5 copies for the 302695 and 4120753 sites. Based on the developed IC-qPCR method, the modification frequency of four PTs in *E*. *coli B7A* was determined with high accuracy, and the results showed that the PT modification was partial and that the modification frequency varied among investigated PT sites. All these results showed that IC-qPCR was suitable for evaluating the PT modification, which would be helpful to further understand the biological function of PT modification.

## Introduction

Microbial epigenetics involves modifications to the nucleosides^1^. Two DNA modification systems, methylation and phosphorothioation (PT), often function as a component of restriction-modification(R-M) systems in bacteria^2–6^. DNA methylation is a common mechanism of epigenetic regulation that occurs by adding a methyl group to DNA molecule, which affects many biological processes. DNA PT modification, where a non-bridging oxygen atom is replaced by a sulfur atom, was discovered in many taxonomically unrelated bacteria^7^. Previous studies have demonstrated that PT modifications are governed by a large family of the five-gene *dndA-E* cluster^8,9^. Many researches were carried out regarding the biological function of Dnd proteins^10–14^. Several approaches have been developed to quantify DNA methylation, such as Sodium bisulfite sequencing method, single molecule real time (SMRT) sequencing, and methylation-specific PCR^15,16^. Sodium bisulfite sequencing method has become the most common technology for the quantitative analysis of DNA methylation. After treated with sodium bisulfite, unmethylated cytosine residues will be converted to uracil, but 5-methylcytosine remains nonreactive. During PCR amplification, unmethylated cytosines appear as thymine and methylated cytosines remain as cytosines allowing 5-methylcytosine to be distinguished from unmethylated cytosines^17–19^. SMRT sequencing was a good tool and used to determine the DNA methylation patterns in bacteria and archaea at whole genome scale^20,21^. The PCR and sequencing based methods are powerful techniques to observe the biological significance of DNA methylation^22^.

To fully understand the PT modification and its biological function, understanding of the PT sites and their frequency at whole genome level is very important. Several methods were developed to reveal the PT modifications in bacterial genome. One selective fluorescent analytical method was developed to achieve the quantification of total PT contents in whole bacterial DNA and found about 455 PTs per million DNA molecules^23^. A liquid chromatography-coupled mass spectrometry (LC-MS/MS) method was developed to quantify the PT modified molecules in prokaryotic genomes, providing a rich source of information about the biological function of PT modification, which showed that the PT modification widely occurs in prokaryotes with different frequency and diverse sequence contexts^7^. Previous works showed that 2-iodoethanol was effective in degrading phosphorothioate-containing DNA and the mechanism of phosphorothioate alkylation and cleavage was also been described^24,25^. Upon iodine treatment a PT modified DNA molecule gets split at the modified site, whereas an unmodified DNA molecule remains intact. Thus, PT modified DNA molecules and unmodified molecules can be identified using DNA amplification methods with designed primers containing the cleavage site. One the basis of the advantages of PCR and sequencing techniques in DNA methylation analysis, SMRT sequencing and deep sequencing combined with iodine-induced cleavage methods were also developed to quantitatively analyze the PTs profile map in bacterial genome^26^. According to the results from SMRT method, 12% of possible 40701 GAAC/GTTC sites in *E*. *coli B7A* genome were found to be modified, which suggested partial modification in these PT sites^26^. However, most of previous work focused on the quantification of total PTs in bacterial genome, while the rules of PT modification at specific site is still unknow. Comprehensive understanding of the function of DNA PT modification requires not only consideration of distribution of PT across the whole genome, but also the details of the PT modification at each site.

In order to quantify the accurate frequency of PT modification at individual site and reveal the potential function of partial PT modification in bacterial genome, we developed one novel method, iodine-induced cleavage quantitative real-time PCR (IC-qPCR), through the combination of TaqMan real-time PCR with the iodine-induced cleavage. Using IC-qPCR, we successfully quantified the accurate PT modification frequency of four PT modified sites in *E*. *coli B7A*, which would be helpful to further understand the rules of PT modification in bacteria.

## Results

### The principle of IC-qPCR in PT modification frequency quantification

**Figure 1:**
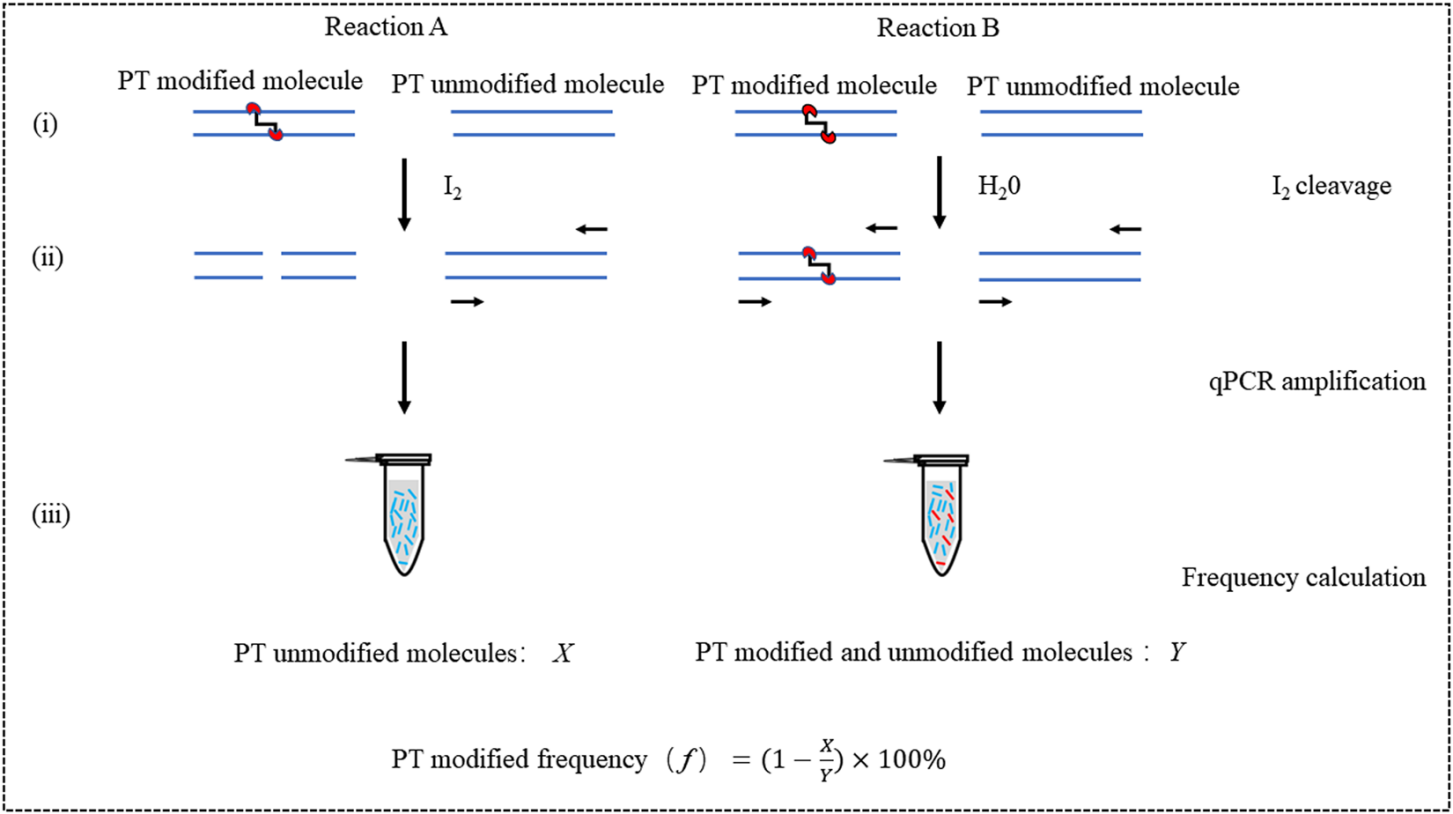
Schematic diagram of the IC-qPCR in the quantification of PT modification frequency. (i) Left (Reaction A): DNA sample was treated with iodine, Right (Reaction B): DNA sample was treated with H_2_O instead of iodine. (ii) The treated DNAs were quantified by real-time PCR analysis, (iii) PT modification frequency of each PT site was calculated according to the formula.

Genomic mapping of phosphorothioates reveals partial modification of short consensus sequences, but the detailed modification frequency at specific site remains unknown. For this aim, we developed a novel method named with IC-qPCR, which consisted of iodine-induced specifically cleavage at PT sites and quantitative real-time PCR. IC-qPCR analysis included three steps as follows (Fig. 1): (i) the tested bacterial genomic DNA was cleaved with Iodine (A) and H_2_O instead of Iodine (B), respectively. (ii) the cleaved DNA products were amplified and quantified in real-time PCR, respectively. In the reaction employing the Iodine treated products as templates, the number of all the genomic DNA molecules without PT modification was quantified as X. In the reaction with the H_2_O treated products, the number of all the genomic DNA molecules, including PT modification and non-PT modification, was quantified as Y; (iii) the PT modification frequency (*f*) was calculated with the formula of $f=(1-\frac{X}{Y})\times 100 \%$.

### Construction of standard curves

**Figure 2:**
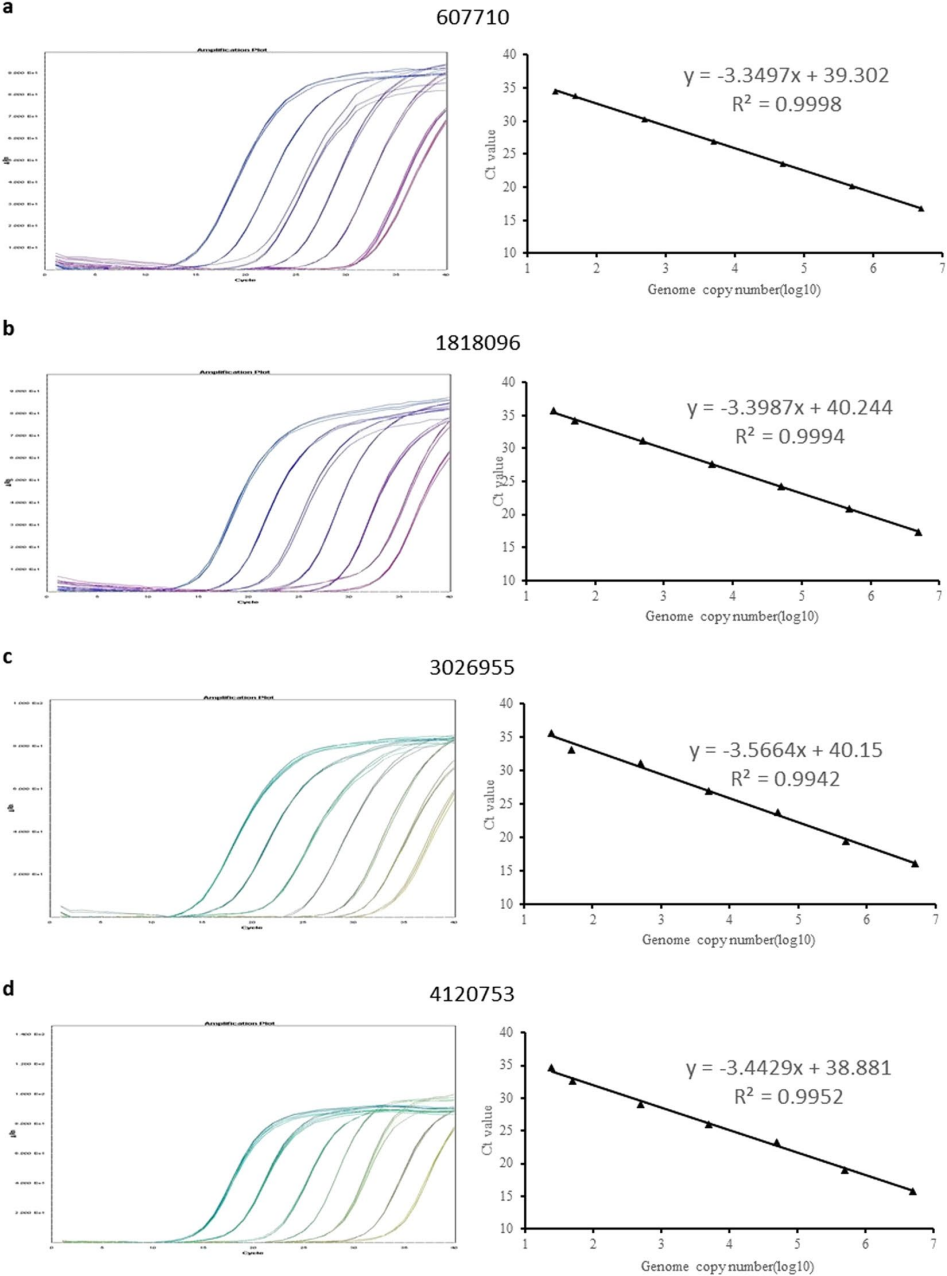
Amplification plots and standard curves of four IC-qPCR assays using *E*. *coli B7A* DNA calibrators ranged from 5 to 1 × 10^6^ copies per reaction. (**a**) 607710 assay, (**b**) 1818096 assay, (**c**) 3026955 assay, (**d**) 4120753 assays. Each reaction was performed in quintuplicates.

A dilution series ranged from 10^6^ to 5 copies per reaction of *E*. *coli B7A* genomic DNA was prepared, and used as calibrators to generate four standard curves for evaluating the PCR efficiency, linearity, and further absolute quantification. The Ct values were plotted against the log (DNA copy numbers) to generate standard curves (Fig. 2). PCR amplification efficiency (E) was calculated according to the equation of E = 10^(−1/slope)^ − 1. The PCR efficiency of four IC-qPCR assays ranged from 91% to 97%. The standard curve of each assay between log of dilution series and Ct values was found with good linearity in a wide range from 10^6^ to 5 copies per reaction. The R^2^ values of the four constructed standard curves were all above 0.99 (ranging from 0.9942 to 0.9998), suggested that all the standard curves had good linearity. The wide dynamic range, high PCR efficiency, and good linearity indicated that the established IC-qPCR assays could be used for quantification of PT modification.

### Sensitivity of IC-qPCR

The limit of quantification (LOQ) refers to the minimum concentration that can be reliably measured with reasonable certainty at 95% confidence level^27^. To evaluate the sensitivity of IC-qPCR assay, dilutions of extracted *E*. *coli B7A* DNA (i.e., 100, 10, 5, and 2 copies/reaction) were tested with fifteen replicates each (As shown in Table 1). In the test, the Ct values decreased as the increase of tested DNA amounts, and Ct values with <35 and lower relative standard deviation (RSD) of ≤25% were observed in all fifteen repeats with 10 copies tested DNAs in all four arrays, respectively. In the IC-qPCR arrays of the 3026955 and 4120753 site, the DNA samples with 5 copies per reaction were quantified in all fifteen repeats. The mean copy number was 6.62 and 4.99 with the bias of 32.37% and −0.20%, respectively. Concluded from the obtained data of copy number, RSD, and bias, the LOQ of developed IC-qPCR was determined as 10 copies per reaction for the 607710 and 1818096 sites and 5 copies for the 3026955 and 4120753 sites. The evaluated LOQ showed the high sensitivity of IC-qPCR arrays, indicating that the developed IC-qPCR could be used for quantification of the PT modification, even for the PT modification with a low frequency.

**Table 1:** Sensitivity of IC-PCR assays for the dilution series of *E*. *coli B7A* DNA.

Array	Copies/reaction	Number of positive	Mean Ct values	Amount copy number	RSD (%)	Bias (%)
607710	100	15/15	30.28	99.05	1.43	−0.95
	10	15/15	33.33	12.13	12.99	21.30
	5	8/15	/	/	/	/
	2	0/15	NA	NA	NA	NA
	100	15/15	31.16	94.24	7.40	−5.76
1818096	10	15/15	34.29	11.28	19.27	12.80
	5	3/15	/	/	/	/
	2	0/15	NA	NA	NA	NA
	100	15/15	30.73	87.57	5.85	12.43
3026955	10	15/15	33.82	11.91	3.29	11.91
	5	15/15	34.73	6.62	9.91	32.37
	2	0/15	NA	NA	NA	NA
	100	15/15	29.45	109.72	3.93	9.72
4120753	10	15/15	32.84	11.37	12.48	13.70
	5	15/15	34.07	4.99	23.46	−0.20
	2	2/15	/	/	/	/

“/” Means no enough data for further analysis, “NA” means no amplification.

### Reproducibility

Reproducibility refers to the results variation of analytical method among different environment and operators, and which is typically evaluated and expressed as the relative standard deviation (RSD) of quantified results^27^. The reproducibility of IC-qPCR assays was determined by assessing the variation of Ct values obtained from 10-folds diluted DNAs (ranging from 1 × 10^6^ to 10 copies per PCR reaction (As shown in Table 2). The RSD values obtained from tested DNA samples ranged from 0.24 to 1.70% for the 607710 assay, 0.19 to 1.27% for the 1818096 assay, 0.13 to 1.40% for the 3026955 assay, and 0.10 to 0.61% for the 4120753 assay. The obtained values indicated that the established IC-qPCR assays could provide creditable quantification of the PT modified within a wide dynamic range.

**Table 2:** Reproducibility, coefficient of variation for qPCR.

Copy number	607710		1818096		3026955		4120753	
	Mean Ct value	SD	Mean Ct value	SD	Mean Ct value	SD	Mean Ct value	SD
1 × 10^6^	16.84	0.05	17.39	0.22	16.20	0.02	15.83	0.09
1 × 10^5^	20.23	0.07	20.91	0.04	19.45	0.11	19.04	0.04
1 × 10^4^	23.51	0.40	24.33	0.14	23.83	0.33	23.28	0.14
1 × 10^3^	26.96	0.11	27.68	0.10	26.99	0.20	26.06	0.05
1 × 10^2^	30.29	0.07	31.21	0.10	31.06	0.19	29.01	0.06
1 × 10^1^	33.77	0.12	34.14	0.29	33.09	0.04	32.61	0.03

### Quantification of PT modification frequency of *E*. *coli B7AΔB-H*

In order to confirm the strategy of IC-qPCR in PT modification quantification, *E*. *coli B7AΔB-H* genome DNA with no PTs was prepared, and used as negative control in the IC-qPCR assay of 607710 site, 1818096 site, 3026955 site, and 4120753 site, respectively. The PT modification frequency of *E*. *coli B7AΔB-H* sample was determined to be −0.95% at the 607710 site, −0.32% at the 1818096 site, 1.06% at the 3026955 site, and −0.02% at the 4120753 site (As shown in Table 3). The obtained results were consistent with expected data (0%), suggesting that this method is accurate enough and can be used for further quantitative analysis of PT modification.

**Table 3:** PT modification frequency of *E*. *coli B7AΔB-H* determined by qPCR.

Assay	X	Y	PT Frequency (*f*, %)
607710	366670	363222	−0.95
1818096	406426	405142	−0.32
3026955	310171	313506	1.06
4120753	419827	419744	−0.02

### Quantification of PT modification frequency in bacterial *E*. *coli B7A*

After being passed the evaluation of specificity, sensitivity, and reproducibility, the novel established IC-qPCR assays were employed to quantify the PT modification frequency in bacterial *E*. *coli B7A*. The IC-qPCR assays were tested in triplicate on three separate days (total of nine results per site). The PT modification frequency were showed in Table 4. The results showed that the PT modification frequency of the 607710 site was 41.31% ± 0.013. Similarly, for the 1818096 site, the mean values was 23.81% ± 0.034. For the 3026955 PT site, the mean value was 8.51% ± 0.006. In the 4120753 PT site analysis, the mean value was determined to be 15.79% ± 0.007. All these values were within the dynamic range of IC-qPCR and were creditable with lower variations. The PT modification frequency in the four sites were no more over 50%, indicating that the PT modification were partial modification in *E*. *coli B7A* and the modification frequency varied among different PT sites.

**Table 4:** PT modification frequency of *E*. *coli B7A* determined by qPCR.

Array	X			Y			Mean PT modification frequency (*f*, %)	SD
	Day 1	Day 2	Day 3	Day 1	Day 2	Day 3		
607710	119569	32747	43514	200454	55362	75984	41.31	0.013
1818096	718292	53239	43252	943438	66871	59392	23.81	0.034
3026955	431269	456215	32331	471407	502230	35092	8.51	0.006
4120753	103968	103023	83264	123108	121519	99841	15.79	0.007

## Discussion

We described a highly accurate quantification method for rapid evaluating PT modification frequency at individual site in bacterial genome DNA, which showed high specificity, wide dynamic range, and high LOD comparing. Previous studies were mainly used for direct analysis of total PT contents of whole genome DNA^7,23,26^. However, IC-qPCR method over the previous one in quantitatively analysis of the unique PT site. The results from the specificity test confirmed that IC-qPCR method was DNA sequence specific and could identify the unique PT site without no cross interaction with other PT sites in whole genome.

The wide dynamic range between 10^^6^ to 10 copies per reaction indicated that IC-qPCR method could be used for quantifying the PT modified DNA molecules of bacterial with different concentrations, which might avoid the variation from DNA samples dilution. The high sensitivity of IC-qPCR method with the LOQ of 5 copies or 10 copies per reaction suggested that the IC-qPCR method was suitable for the quantification of PT modification with a low frequency. The results of *E*. *coli B7A* DNA test showed that all four PT sites were partial modification and that the PT modification frequency varied from 41.31% to 8.51%. One previous study employing SMRT sequencing method reported the PT sites and their modification of *E*. *coli B7A* at whole genome level, and the results showed that the PT modification were observed only at 607710 and 1818096 sites and non-PT modification happened at 3026955 and 4120753 sites^26^. The results of IC-qPCR at 607710 and 1818096 sites in this study were consistent with SMRT results. However, PT modification was observed and quantified at 3026955 and 4120753 sites by IC-qPCR analysis, which was completely different from those of SMRT analysis. The SMRT sequencing platform uniquely detects DNA modifications by monitoring the interpulse duration (IPD), and the IPD refers to the average signal of all DNA molecules. In SMRT analysis, low PT modification frequency often leads to low IPD values, and the signal cannot be clearly distinguished from the background, which will induce the miss or underestimate the low PT modification^28–30^. However, the IC-qPCR could estimate the low PT modification and avoid missing and underestimating the low PT modification because of the wide dynamic and high sensitivity. The PT modification frequency with slightly low value of 8.51% and 15.79% at the 3026955 and 4120753 sites was well evaluated.

In conclusion, the developed IC-qPCR method could achieve the quantification of PT modification frequency in different levels, even for PT modification with a low frequency. Based on the results of IC-qPCR analysis, we also confirmed our previous result that the PT modification in bacteria was partial and often varied among different PT sites, and the observations of PT modified rules at single site would be helpful to further understand the biological function of PT modifications in bacteria.

## Materials and Methods

### Materials and bacterial strains

*E*. *coli B7A* was obtained from Dr Jaquelyn Fleckenstein (Departments of Medicine and Molecular Sciences, University of Tennessee Health Science Center)^31^. *E*. *coli B7A* possesses *dndB-H* genes and with modification at the G_ps_AAC/G_ps_TTC motif, while *E*. *coli B7AΔB-H* was a *dndB-H* gene deleted mutant strain which was previously constructed in our lab(Supplementary Table S1)^7,26^. In this work, the *E*. *coli B7AΔB-H* was used as negative control for IC-qPCR analysis. Bacterial DNA Kit purchased from TIANGEN (TIANGEN, Cat. no. DP302-02) was used to isolate and purify the *E*. *coli B7A* and *E*. *coli B7AΔB-H* genomic DNA. Extracted DNA quantity and quality were evaluated and measured with Spectrophotometer Nanodrop-1000 (Thermo Fisher Scientific, Waltham, MA, USA). Water was deionized and filtered using a MilliQ water purification system (EMD Millipore, Billerica, MA).

### Primers and TaqMan probes

Our previous studies indicated that 4855 out of 40,701 GAAC/GTTC sites were modified in *E*. *coli B7A* genome^26^. Based on these results, four modified sites (607710 site, 1818096 site, 3026955 site, and 4120753 site) were selected as the targets of developed IC-qPCR assay in this study (Supplementary Tables S2 and S3). The primers and probes for four sites were designed based on the specific DNA sequences using Beacon Designer 8.0 and listed in Table 5. The TaqMan probes were labeled with 6-carboxyfluorescein (FAM) at the 5′ end and black hole quencher (BHQ I) at the 3′ end. The primers and probes were synthesized and purchased from Sangon Biotech, Co., Ltd. (shanghai, China).

**Table 5:** Primers/probes for real-time PCR.

Site	Genome position	Primer/probes	Primer sequence (5′-3′)	Product size (bp)
G1	607710	G1-F	GATGGCTTCGTTAAGTGTTAGTCC	109
		G1-R	GCTGACTCTGACATTATGGTATCG	
		G1P	CCTGTTCTCACCGCATGGTCAACGCC	
G2	1818096	G2-F	ACGCAATTCACGTACTGACATG	124
		G2-R	AAAAGCCAAACTTTTCGAATTAATGAC	
		G2P	ATCAACATCGTCAAGCGTCATGCCGG	
G3	3026955	G3-F	ATCAGAGAGAGAAGACCGAAACC	119
		G3-R	CCACGAGTACACCTCTCCTTAG	
		G3P	TCATCGTGAATCCATTAGACTTAGAAAATATCGGGTC	
G4	4120753	G4-F	TAAAGATGGATGGGCAGATCGG	144
		G4-R	GGATCACGAAAAGTATCTCTGGAC	
		G4P	CGCCTCGTTCGCCTTTGCCGCC	

### Iodine cleaved treatment of *E*. *coli B7A* DNA

Iodine solution in ethanol was freshly prepared for use. The Iodine cleavage reaction was performed with a final volume of 100 μl, including 20 μg *E*. *coli B7A* genomic DNA, 50 mM Na_2_HPO_4_ (PH 9.0), and 3 mM Iodine or 10% ethanol (used in control reaction). The reaction parameters were as follows: 65 °C for 10 min, and lowered the temperature slowly to 4 °C (0.1 °C s^−1^). After the Iodine treatment, the cleavage reaction products were used as templates for further real-time PCR analysis.

### Real-time PCR quantification assays and data analysis

The iodine cleaved DNA and control DNA were used as DNA templates in real-time PCR reactions. Real-time PCR was carried on ABI7900 real-time PCR system (Applied Biosystems, USA). The real-time PCR was performed with a final volume of 25 µl, including 12.5 µl of 2 × HR qPCR Master Mix, 1 µl of 10 μM forward primer, 1 µl of 10 μM reverse primer, 0.5 µl of 10 μM probes, 5 µl of DNA template, and 5 µl of DNAse free water. The real-time PCR was run with following program: 95 °C for 10 min, followed by 40 cycles of 15 s at 95 °C and 1 min at 60 °C. The fluorescent signal was monitored in extension step of each cycle. Wells with Ct values higher than 35 were considered as negative. In real-time PCR analysis, the reaction employing ddH_2_O as templates was used as a no template control (NTC). Each reaction was performed with three repeats in three different days, and each repeat with three parallels. The SDS 2.4 software (Applied Biosystems, USA) was used for statistical analyses. Data were further exported to Micro Excel for further analysis. The statistical standardized curves of all real-time PCR assays were constructed using serial dilutions of *E*. *coli B7A* genome DNA as calibrators. The copy number of tested samples were calculated according to the constructed standard curves.

## Supplementary information

Supplementary Table S1-S3

## Data Availability

All data generated or analyzed during this study are included in this published article (and its Supplementary Information files).

## References

[1] Casadesus J, Low DA (2006). Epigenetic gene regulation in the bacterial world. J. Microbiol Mol Biol Rev..

[2] Chen C (2017). Convergence of DNA methylation and phosphorothioation epigenetics in bacterial genomes. J. Proc Natl Acad Sci USA.

[3] Moore LD, Le T, Fan G (2013). DNA methylation and its basic function. J. Neuropsychopharmacology..

[4] Meselson M, Yuan R, Heywood J (1972). Restriction and modification of DNA. J. Annu Rev Biochem..

[5] Xu T, Yao F, Zhou X, Deng Z, You D (2010). A novel host-specific restriction system associated with DNA backbone S-modification in Salmonella. J. Nucleic Acids Res..

[6] Cao B (2014). Pathological phenotypes and *in vivo* DNA cleavage by unrestrained activity of a phosphorothioate-based restriction system in Salmonella. J. Mol Microbiol..

[7] Wang L (2011). DNA phosphorothioation is widespread and quantized in bacterial genomes. J. Proc Natl Acad Sci USA.

[8] Zhou Xiufen (2005). A novel DNA modification by sulphur. J. Mol Microbiol..

[9] Xu T. *et al*. DNA phosphorothioation in Streptomyces lividans: mutational analysis of the dnd locus. *J*. *BMC Microbiol*. **9** (2009). 10.1186/1471-2180-9-41 PMC2653506 19232083

[10] Zheng T (2016). DndEi exhibits helicase activity essential for DNA phos-phorothioate modi-fication and ATPase activity strongly stimulated by DNA substrate with a GAAC/GTTC motif. J. Biol Chem..

[11] Cao B. *et al*. *In vitro* analysis of phosphorothioate modification of DNA reveals substrate recognition by a multiprotein complex. *J*. *Sci Rep*. **5** (2015). 10.1038/srep12513 PMC4515589 26213215

[12] Cheng Q (2015). Regulation of DNA phosphorothioate modifications by the transcriptional regulator DptB in Salmonella. J. Mol Microbiol..

[13] You D, Wang L, Yao F, Zhou X, Deng Z (2007). A novel DNA modification by sulfur: DndA is a NifS-like cysteine desul-furase capable of assembling DndC as an iron-sulfur cluster protein in Streptomyces lividans. J. Biochemistry..

[14] Yao F, Xu T, Zhou X, Deng Z, You D (2009). Functional analysis of spfD gene involved in DNA phosphorothioation in Pseudomonas fluorescens Pf0-1. J. FEBS Lett..

[15] Jones PA (2012). Functions of DNA methylation: islands, start sites, gene bodies and beyond. J. Nat Rev Genet..

[16] Redshaw, N., Huggett, J. F., Taylor, M. S., Foy, C. A. & Devonshire, A. S. Quantification of epigenetic biomarkers: an evaluation of estab-lished and emerging methods for DNA methylation analysis. *J*. *BMC Genomics*. **15** (2014). 10.1186/1471-2164-15-1174 PMC4523014 25539843

[17] Frommer M (1992). A genomic sequencing protocol that yields a positive display of 5-methylcytosine residues in individual DNA strands. J. Proc Natl Acad Sci USA.

[18] Patterson, K., Molloy, L., Qu, W. & Clark, S. DNA Methylation: bisulphite modification and analysis. *J*.*Vis Exp*. **56** (2011). 10.3791/3170 PMC3227193 22042230

[19] Cokus SJ (2008). Shotgun bisulphite sequencing of the Arabidopsis genome reveals DNA methylation patterning. J. Nature..

[20] Blow, M. J. *et al*. The epigenomic landscape of prokaryotes. *J*. *PLoS Genet*. **12** (2016). 10.1371/journal.pgen.1005854 PMC4752239 26870957

[21] Chen P, Jeannotte R, Weimer BC (2014). Exploring bacterial epigenomics in the next-generation sequencing era: a new approach for an emerging frontier. J. Trends Microbiol..

[22] Harris RA (2010). Comparison of sequencing-based methods to profile DnA methylation and identification of monoallelic epigenetic modifications. J. Nat Biotechnol..

[23] Xiao L, Xiang Y (2016). Quantification of total phosphorothioate in bacterial DNA by a bromoimane-based fluorescence method. J. Biotechnol J..

[24] Gish G, Eckstein F (1988). DNA and RNA sequence determination based on phosphorothioate chemistry. J. Science..

[25] Nakamaye KL (1988). Direct sequencing of polymerase chain reaction amplified DNA fragments through the incorporation of deoxynucleoside alpha-thiotriphosphates. J. Nucleic Acids Res..

[26] Cao, B. *et al*. Genomic mapping of phosphorothioates reveals partial modification of short consensus sequences. *J*. *Nat Commun*. **5** (2014). 10.1038/ncomms4951 PMC4322921 24899568

[27] Bustin SA (2009). The MIQE guidelines: minimum information for publication of quantitative real-time PCR experiments. J. Clin Chem..

[28] Flusberg BA (2010). Direct detection of DNA methylation during single-molecule, real-time sequencing. J. Nat Methods..

[29] Korlach J (2010). Real-time DNA sequencing from single polymerase molecules. J. Methods Enzymol..

[30] Song CX (2011). Sensitive and specific single-molecule sequencing of 5-hydroxymethylcytosine. J. Nat Methods..

[31] Wang L (2007). Phosphorothioation of DNA in bacteria by dnd genes. Nat Chem Biol..

