# Supplementary material for: Novel Iodine-induced Cleavage Real-time PCR Assay for Accurate Quantification of Phosphorothioate Modified Sites in Bacterial DNA
Source: Sci Rep. 2019 May 16;9:7485. doi: 10.1038/s41598-019-44011-x (PMC6522622; doi:10.1038/s41598-019-44011-x)
Supplement: Supplementary file 1 — Supplementary Table S1-S3 [file 41598_2019_44011_MOESM1_ESM.docx]

**Novel Iodine-induced Cleavage Real-time PCR Assay for Accurate Quantification of Phosphorothioate Modified Sites in Bacterial DNA**

*Yi Chen^a^, Tao Zheng^a^, Jinli Li^a^, Jinjie Cui^b^, Zixin Deng^a^, Delin You^a*^, Litao Yang^a,b*^*

**Supplementary Tables**

**Supplementary Table S1. Strains used in this study**

| **Strain** | **Characteristics** | **Source** |
| --- | --- | --- |
| *E. coli* B7A | PT^+^, O148:H28:CS6:LT^+^:ST^+^ | This work |
| *E. coli* B7A △*dndB-H* | PT^-^, *dndB-H* genes deficient in B7A | This work |

**Supplementary Table S2. The position and characteristics of 4 Loci selected for analysis**

| **Genome position** | **Type** | **Function** | **Subsystems** | **Detected in SMRT sequencing** |
| --- | --- | --- | --- | --- |
| 607710 | Gene | benzoate transporter | Secondary metabolites biosynthesis, transport, and catabolism | + |
| 1818096 | Gene | Gp41 | Glycoprotein | + |
| 3026955 | Promoter | Rep | ATP-dependent DNA helicase | - |
| 4120753 | Gene | hypothetical protein | Function unknown | - |

**Supplementary Table S3. The DNA sequence of 4 Loci and their PT modification sites selected for analysis**

| **Genome position** | **Sequence Information** |
| --- | --- |
| 607711 | GATGGCTTCGTTAAGTGTTAGTCCCTGCAATCCGGTGACCAACAAAGCCGCGCCAGGCGTTGACCATGCGGTGAGAACAGGTACGGGCGTTCGATACCATAATGTCAGAGTCAGC |
| 1818097 | ACGCAATTCACGTACTGACATGCGATCAACATCGTCAAGCGTCATGCCGGCAACTGTGCCGCCGTCGGCCAGTTCGGCAAGCTCTTCGTCGTCTTCTGTCATTAATTCGAAAAGTTTGGCTTTT |
| 3026956 | ATCAGAGAGAGAAGACCGAAACCGGAGTTCATCGTGAATCCATTAGACTTAGAAAATATCGGGTCGTAACGTTGTGTTATTACTTTTTCAGAGTTTACTAAGGAGAGGTGTACTCGTGG |
| 4120754 | TAAAGATGGATGGGCAGATCGGCCACTGCGCCACCTGCTTGTGGACCACGGATCTGCACGCACCAGCCATTATTCACAAAGCTCTTCACCATATTGGCGGCAAAGGCGAACGAGGCGTTTGTCCAGAGATACTTTTCGTGATCC |
